# Short- and Medium-Term Exposure to Ocean Acidification Reduces Olfactory Sensitivity in Gilthead Seabream

**DOI:** 10.3389/fphys.2019.00731

**Published:** 2019-07-03

**Authors:** Zélia Velez, Christina C. Roggatz, David M. Benoit, Jörg D. Hardege, Peter C. Hubbard

**Affiliations:** ^1^ Centro de Ciências do Mar, Faro, Portugal; ^2^ Energy and Environment Institute, University of Hull, Hull, United Kingdom; ^3^ Department of Biological and Marine Science, University of Hull, Hull, United Kingdom; ^4^ E.A. Milne Centre for Astrophysics and G.W. Gray Centre for Advanced Material, University of Hull, Hull, United Kingdom

**Keywords:** olfaction, ocean acidification, fish, amino acid, receptor, olfactory epithelium, carbon dioxide, protonation

## Abstract

The effects of ocean acidification on fish are only partially understood. Studies on olfaction are mostly limited to behavioral alterations of coral reef fish; studies on temperate species and/or with economic importance are scarce. The current study evaluated the effects of short- and medium-term exposure to ocean acidification on the olfactory system of gilthead seabream (*Sparus aurata*), and attempted to explain observed differences in sensitivity by changes in the protonation state of amino acid odorants. Short-term exposure to elevated *P*CO_2_ decreased olfactory sensitivity to some odorants, such as L-serine, L-leucine, L-arginine, L-glutamate, and conspecific intestinal fluid, but not to others, such as L-glutamine and conspecific bile fluid. Seabream were unable to compensate for high *P*CO_2_ levels in the medium term; after 4 weeks exposure to high *P*CO_2_, the olfactory sensitivity remained lower in elevated *P*CO_2_ water. The decrease in olfactory sensitivity in high *P*CO_2_ water could be partly attributed to changes in the protonation state of the odorants and/or their receptor(s); we illustrate how protonation due to reduced pH causes changes in the charge distribution of odorant molecules, an essential component for ligand-receptor interaction. However, there are other mechanisms involved. At a histological level, the olfactory epithelium contained higher densities of mucus cells in fish kept in high CO_2_ water, and a shift in pH of the mucus they produced to more neutral. These differences suggest a physiological response of the olfactory epithelium to lower pH and/or high CO_2_ levels, but an inability to fully counteract the effects of acidification on olfactory sensitivity. Therefore, the current study provides evidence for a direct, medium term, global effect of ocean acidification on olfactory sensitivity in fish, and possibly other marine organisms, and suggests a partial explanatory mechanism.

## Introduction

Since the Industrial Revolution, atmospheric carbon dioxide (CO_2_) levels have been steadily increasing and the oceans of the world act as a sink for much of this. As atmospheric CO_2_ has increased from pre-industrial (about 280 μatm CO_2_) to present day values (about 390 μatm CO_2_) (Doney et al., 2009), this has led to a fall in oceanic pH of 0.1 unit, with a further fall of 0.77 units predicted by the year 2300 (Doney et al., 2009). Global mean oceanic CO_2_ levels are expected to reach 1,000 μatm by the year 2100, and 1900 μatm CO_2_ by 2300 (Caldeira and Wickett, 2003). Studies prior to 2009 suggested that fish, as effective acid-base regulators, were not particularly sensitive to changes in *P*CO_2_. However, more recent studies have documented notable impacts on neurosensory and behavioral endpoints in fish at *P*CO_2_ levels predicted to occur before the end of the 21st century (reviewed in: Heuer and Grosell, 2014; Clements and Hunt, 2015; Ashur et al., 2017). Consistent disturbances have been noted across a range of sensory systems, including olfaction (Dixson et al., 2010; Cripps et al., 2011; Devine et al., 2012a,b), hearing (Simpson et al., 2011), and vision (Chung et al., 2014), and have also been implicated in processes linked to general cognitive function including changes in lateralization (Domenici et al., 2012) and learning (Ferrari et al., 2012). The implications of these disturbances are expected to be substantial and include changes to dispersal, recruitment, connectivity, social interactions, predator-prey dynamics, population replenishment, biodiversity, habitat preference, and settlement timing; all of which could drastically affect population and ecosystem dynamics (Heuer and Grosell, 2014).

Evidence suggests that predicted end-of-the-century *P*CO_2_ levels will have adverse effects on the olfactory-mediated behavior of most fish species, including reef species (Dixson et al., 2010; Munday et al., 2016a,b), temperate species (Jutfelt et al., 2013), and sharks (Green and Jutfelt, 2014; Dixson et al., 2015). For example, clownfish larvae exposed to 1,000 μatm CO_2_ did not respond appropriately to the odor of predatory fishes (Dixson et al., 2010); moreover, juvenile damselfish exposed to increased *P*CO_2_ and then released into the wild suffered an 8-fold increase in predation (Munday et al., 2010). In both studies, control juveniles avoided predator odors, whereas CO_2_-treated juveniles were attracted to them (Dixson et al., 2010; Munday et al., 2010), suggesting that mal-adaptive behavioral responses may result from altered sensory processing by the central nervous system. An important step in understanding the basis for neurosensory disruption in fish was reported by Nilsson and colleagues in 2012, who demonstrated that the GABA and GABA_A_ receptors were associated with disruptions in olfaction and lateralization during CO_2_ exposure (Nilsson et al., 2012). The addition of gabazine, a GABA_A_ receptor antagonist, was found to re-establish adaptive olfactory-mediated behavior and lateralization in CO_2_-exposed fish (Nilsson et al., 2012). Since then, other studies have demonstrated the ability of gabazine to restore much of the high *P*CO_2_ related altered behaviors (Chivers et al., 2014; Hamilton et al., 2014; Lai et al., 2015). To date, disruption in brain neurotransmitter function has been proposed as the main mechanism involved in the behavioral effects of high CO_2_ in fish (critically reviewed by Tresguerres and Hamilton, 2017). However, the direct effect of high *P*CO_2_/low pH on the olfactory system may have been underestimated. Recently, acute exposure to acidified seawater was shown to directly reduce olfactory sensitivity of the sea bass (*Dicentrarchus labrax*) to some odorants (Porteus et al., 2018); however, the mechanism remains unknown. In addition, neither medium- or long-term effects on sensitivity were not investigated, nor whether the olfactory system was able to respond at the structural level and counteract the effects of acidification. Assessing the occurrence of morphological and/or physiological alterations in response to increased *P*CO_2_ is crucial to predict the effects of ocean acidification in fish. Therefore, the main objective of the current work was to evaluate the effects of both acute and longer-term exposure to low pH/high *P*CO_2_ on the olfactory sensitivity of the gilthead bream (*Sparus aurata*), an economically important species. The observed effects on olfactory sensitivity were considered in the context of predicted effects of pH change on the protonation of odorant molecules. Finally, we evaluated whether exposure to high *P*CO_2_ provoked structural changes in the olfactory epithelium that might be indicative of adaptation of the olfactory epithelium to lower water pH.

## Materials And Methods

### Fish Maintenance

Animal maintenance and experimentation were carried out in certified experimental facilities and followed Portuguese national legislation (DL 113/2013) under a “group-1” license by the Veterinary General Directorate, Ministry of Agriculture, Rural Development and Fisheries of Portugal. Gilthead seabream (*S. aurata*: 180 ± 7 g; 22.8 ± 0.6 cm), hereafter “seabream,” were obtained from a commercial supplier (Maresa–Mariscos de Esteros, SA, Huelva, Spain) and maintained in 1,000 L tanks with continuously running natural seawater, under natural photoperiod and temperature, and fed with commercial pellets (Sparos, Olhão, Portugal). For experimental purposes, seabream were randomly distributed into six 100 L tanks in an open circuit system kept at natural temperature and photoperiod. Three tanks were kept in control conditions (“control fish”), while the other three were kept at low pH by bubbling CO_2_ gas (see below) into the water and maintained for 4–8 weeks before electrophysiology experiments (“high CO_2_ fish”). No mortality was observed during this time.

### Seawater Chemistry

Seawater was pumped from the ocean into two (2,000 L) header tanks, where it was aerated with ambient air (control) or CO_2_ to achieve the desired pH (elevated-CO_2_ treatment). The *P*CO_2_ of the CO_2_ treatment header tank was maintained at the target value of 1,000 μatm using a pH probe connected to an internal controller (EXAxt PH450G, Yokogawa Iberia, Portugal).

Control and high CO_2_ seawater was supplied to 100 L tanks at 2 L min^−1^. Seawater pH (Orion star A221, Thermo Scientific, Portugal), temperature (Orion star A221, Thermo Scientific, Portugal), and salinity (WTW, cond3310, Spain) were recorded daily in each tank. Water samples were analyzed for total alkalinity twice a week by Gran titration (DL15 titrator, Mettler Toledo, Portugal) using a certified acid titrant (0.1 M HCl, Fluka Analytical, Sigma-Aldrich). Carbonate chemistry parameters (Table 1) were calculated in CO2SYS (Pierrot et al., 2006) using the constants K1 and K2 from Mehrbach et al. (1973) refit by Dickson and Millero (1987) and Dickson (1990) for KHSO_4_.

**Table 1:** Water chemistry parameters for control and elevated CO_2_ tanks.

Parameter	Control CO_2_	High CO_2_
pH_NBS_	8.184 ± 0.004	7.813 ± 0.001
Temperature (°C)	17.0 ± 0.2	18.4 ± 0.3
Salinity (ppt)	34.6 ± 0.02	34.6 ± 0.02
Total Alkalinity (μmol/kg SW)	2,511 ± 15	2,531 ± 21
*p*CO_2_ (μatm)	349 ± 19	1,039 ± 49

Data are given as mean ± SEM. The slight difference in temperature was caused by different exposure to the sun of the header tanks (temperatures were measured at 14:30).

### Selection of Odorants

Odorant, in the context of the current study, can be defined as any chemical detected by the olfactory system which, in vertebrates, is the chemosensory system associated with cranial nerve I (discussed in Mollo et al., 2014, 2017). The effect of acute (minutes) and medium-term (4 weeks) exposure to high *P*CO_2_ on olfactory sensitivity was tested for amino acids, bile fluid, and intestinal fluid. Olfactory sensitivity to amino acids in fish is believed to be mainly involved in food detection (Hara, 1994; Velez et al., 2007). Bile fluid and intestinal fluid are known to be potent olfactory stimuli in fish, possibly involved in chemically-mediated interactions both within and between species (Velez et al., 2009; Huertas et al., 2010; Buchinger et al., 2014; Fatsini et al., 2017; Hubbard et al., 2017). Therefore, we tested the effect of elevated CO_2_/low pH on the olfactory responses of seabream to conspecific bile, intestinal fluid, and a range of amino acids in fish maintained under control conditions (pH nominally 8.2; *P*CO_2_ nominally 350 μatm) and high CO_2_ conditions (pH nominally 7.7, *P*CO_2_ nominally 1,000 μatm) for 4–8 weeks.

### Collection of Body Fluids

Body fluids (bile and intestinal fluid) were collected from five seabream (144 ± 16 g; 18.7 ± 1.0 cm; all males) kept in normal seawater. Fish were anesthetized with 2-phenoxy ethanol (Sigma-Aldrich, Portugal; 2.0 ml L^−1^) and quickly sacrificed by decapitation. Bile fluid was taken directly from the gall bladder and the intestinal fluid was extracted from the posterior 10 cm of intestine. Samples were pooled, diluted in distilled water (1:2), mixed thoroughly, centrifuged, aliquoted, and frozen (−20°C) until use.

### Olfactory Nerve Recording

Seabream were anesthetized in aerated natural seawater containing 300 mg L^−1^ MS222 (ethyl-3-aminobenzoate methanesulfonate salt, Sigma-Aldrich, Portugal) until the response to a tail pinch had stopped; an intramuscular injection of the neuromuscular blocker gallamine triethiodide (Sigma-Aldrich, Portugal; 10 mg/kg in 0.9% NaCl) was then given. Fish were then placed in a padded V-support and the gills flushed with aerated seawater containing 150 mg l^−1^ MS222. The pH of the water flushing the gills was around 8.2 for control fish and 7.7 for fish kept in high CO_2_. Therefore, for control fish, only the olfactory epithelium experienced high CO_2_ conditions during electrophysiology experiments; water chemistry parameters during experiments are summarized in Table 2.

**Table 2:** Water chemistry parameters for control and elevated CO_2_ fish in the electrophysiology set up.

	Control fish		High CO_2_ fish	
	Control water	High CO_2_ water	Control water	High CO_2_ water
pH_NBS_	8.20 ± 0.01	7.70 ± 0.01	8.19 ± 0.01	7.72 ± 0.01
Temperature	22.0 ± 0.2	22.0 ± 0.2	20.4 ± 0.3	20.4 ± 0.3
Salinity	34 ± 0.2	34 ± 0.2	34 ± 0.2	34 ± 0.2
Total alkalinity (μmol/kg SW)	2,562 ± 32	2,575 ± 29	2,648 ± 15	2,655 ± 17
*p*CO_2_	296 ± 12	1,150 ± 23	318 ± 9	1,127 ± 23

Data are given as mean ± SEM.

The olfactory rosette was exposed by cutting the skin and connective tissue overlying the nasal cavity. The nostril was constantly irrigated with filtered sea water (without anesthetic) under gravity (flow rate: 6 ml min^−1^) *via* a glass tube. Test solutions were delivered to the tube irrigating the nasal cavity *via* a computer-operated three-way solenoid valve for 4 s. The olfactory nerves were exposed by removal of the skin, connective tissue, and overlying bone (Hubbard et al., 2000, 2003a). Charcoal-filtered sea water was used to make up the odorants solutions and to irrigate the olfactory rosette during experiments. This water was either bubbled with air (control) or CO_2_ (low pH) until the desired pH_NBS_ was reached. Amino acid solutions were prepared from frozen aliquots of 10^−2^ M; all stimuli were diluted in charcoal-filtered sea water (either control or high CO_2_ as appropriate) immediately prior to use. The order in which odorants were given was randomized, but each odorant was always given from the lowest to the highest concentration.

The electrodes were placed in the olfactory nerve in a position that gave maximal response to 10^−3^ M L-serine. Olfactory nerve activity was recorded using tungsten micro-electrodes (0.1 MΩ, World Precision Instruments, UK) as previously described (Hubbard et al., 2000). Fish were connected to earth *via* a copper wire inserted in the flank. The raw signal was amplified (x20,000; AC pre-amplifier, Neurolog NL104; Digitimer Ltd., Welwyn Garden City, UK), filtered (high pass: 200 Hz, low pass: 3,000 Hz; Neurolog NL125, Digitimer Ltd.) and integrated (time constant 1 s; Neurolog NL703, Digitimer Ltd.). Raw and integrated signals were digitized (Digidata 1440A, Molecular Devices, San Jose, CA, USA) and recorded on a PC running AxoScope™ software (version 10.6, Molecular Devices).

All integrated response amplitudes were normalized to the amplitude of the integrated response to 10^−3^ M L-serine (the “standard”). Responses to the standard were recorded regularly at the beginning and end of each group of samples (every three to five samples) throughout the recording session. Each stimulus was applied for 4 s, with at least 1 min between odorants to allow complete recovery of the receptors.

### Calculation of Amino Acid Protonation State

Odorant molecules, such as amino acids, can be subjected to ionization processes depending on the pH of their surroundings. Amino acids possess two or more ionizable functional groups that are de-protonated at different pH levels. The p*K_a_* value of each ionizable group of a molecule represents the pH at which 50% of the molecules in solution are protonated at this group and 50% remain unchanged. The Henderson-Hasselbalch equation relates pH and the group-specific p*K_a_* values for each amino acid, and therefore calculates the abundance of different protonation states across the entire pH range of interest (for details, see Po and Senozan, 2001, and references therein). At any given pH, a mixture of different protonation states can be present. For the current study, we assumed that the amino acid forms with deprotonated head group to be the form binding to the olfactory receptors. We, therefore, calculated the concentration of this form (= effective concentrations) of different amino acids used in the experiments for the control (pH 8.2) and treatment (pH 7.7) conditions based on the concentration used during the bioassay and the group-specific p*K_a_* values for each amino acid obtained from the CRC Handbook of Chemistry and Physics (Lide, 2004).

### Comparison of pH-Induced Changes in Amino Acid Protonation State to Olfactory Potency

For direct comparison of the change in protonation state and the olfactory response for different concentrations and pH conditions, the respective effective stimulus concentrations were inserted into the linear regression equation/Hill plot obtained from the integrated nerve response at a given pH (see “Data Analysis” for details). The resulting points were plotted in the same figure to visualize the extent of change caused by stimulus protonation vs. the extent of change observed for the nerve response.

### Conformers and Charge Distribution of Different Protonation States by Quantum Chemical Calculations

We used quantum chemical calculations to obtain the energetically most favorable model conformers for the two protonation states of interest and assess differences in their molecular electrostatic potential (MEP), which describes the charge distribution around the molecule. Starting from the structures published by Rak et al. (2001), the protonation state models of L-arginine were optimized using the PBE0 exchange correlation functional (Adamo and Barone, 1999) with a pc-2 basis set (Jensen, 2001, 2002a,b) and water as implicit solvent using COSMO (Klamt and Schüürmann, 1993) implemented in the ORCA suite of programs (Neese, 2012) (Version 3.0.0). We used the RIJ-COSX approximation (Neese et al., 2009) with a def2-TZVPP/J auxiliary basis set (Weigend and Ahlrichs, 2005) and included D3 dispersion corrections following Grimme et al. (2010, 2011). The VeryTightSCF and TightOpt criteria implemented in ORCA were used to stop the SCF gradient and the optimization at a total energy change of <10^−8^ E_h_, respectively. Full optimization was confirmed by ensuring that there were no negative numerical frequencies for each conformer. The calculation of the molecular electrostatic potential (MEP) was performed with the GAMESS program (vJan122009R1) using the PBE exchange correlation functional (Perdew et al., 1996) in conjunction with a STO-3G basis set (Hehre et al., 1969; Collins et al., 1976). Three-dimensional electron density iso-surface was visualized with 100 grid points, a medium grid size, and a contour value of 0.03 e·a_0_^−3^ using the MacMolPlt program (Bode and Gordon, 1998) (v7.5141). The density iso-surface was colored according to the MEP with a maximum value of 0.25 E_h_·e^−1^ and the RGB color scheme with red representing positive, green neutral, and blue negative charge.

### Histology

Seabream were killed rapidly using an anesthetic overdose (500 mg/l MS222). Olfactory rosettes were carefully dissected from 12 fish (six controls, and six in high CO_2_ water for at least 4 weeks) under a stereo-microscope. Olfactory epithelia were fixed in Bouin’s solution (Sigma, Portugal) for 20 h and washed with 70% ethanol. Tissues were then embedded in paraffin and sectioned longitudinally (5 μm sections). Serial sections of the tissues were collected until the complete olfactory organ was visible (including the central raphe). The dewaxed and rehydrated sections were stained with Mayer’s hematoxylin/eosin and neutral and acidic mucus was identified using a combined periodic acid-Schiff (PAS)-Alcian blue stain. Slides were examined under a microscope (Leica DM 2000, Famalicão, Portugal) and photographic images were obtained using a digital camera (Leica DFC 480, IM50-software). For analysis, we focused on the three central lamellae (lamellae 7, 8, and 9) of the olfactory rosette. The ratio between nonsensory vs. sensory epithelium within an olfactory lamella was assessed by dividing the length of the apical nonsensory epithelium by the length of the olfactory lamella (from the top to the central raphe). Images were analyzed using the software Fiji Image J. Statistically significant differences between the ratio of nonsensory vs. sensory epithelium in control animals and experimental animals was assessed using Student’s *t* test (Prism 6). The goblet (mucus producing) cells in the apical surface of the lamellae were quantified using Fiji Image J.

### Data and Statistical Analysis

All statistical analyses of electrophysiological results were carried out on normalized data. To determine whether there were effects of tank pH (i.e., control vs. high CO_2_), odorant pH and concentration, an ANOVA analysis was preformed (SPSS statistics). Factors that showed an effect were further analyzed by linear regression or fit to a three-parameter Hill plot.

Olfactory nerve responses to L-serine, L-leucine, L-arginine, bile fluid, and intestinal fluid were analyzed by linear regression; comparison between nerve responses at different tank, and odorant pH was assessed by linear regression of log-transformed data (Hubbard et al., 2003b) and comparing both the slopes and elevations (Prism 6). The slope of each curve indicates how a given increase in odorant concentration evokes a higher amplitude response; this depends on the binding affinity between ligand (odorant in this case) and receptor. Detection thresholds, which also depend on ligand-receptor affinity, were determined for each independent experiment, from the intercept with the *x*-axis of linear regression fit to individual concentration-response curves for each odorant and each treatment (Prism 6). Differences between detection thresholds were tested using Student’s *t* test for paired data (log). Responses to L-glutamine and L-glutamic acid were described by a three-parameter Hill curve as previously described (Hubbard et al., 2000). EC_50_ and *E*_max_ were calculated for each fish. These data were then compared using Student’s *t*-test (Prism 6).

To evaluate whether normalized data could be used to compare olfactory responses between control and high CO_2_ fish, we first compared raw integrated responses to L-serine at pH 8.2 in control and high CO_2_ fish. There were no differences in the slope (*p* = 0.71) or elevation (*p* = 0.88) of the concentration-response curves to L-serine in control and experimental animals (data not shown); therefore, we compared normalized olfactory nerve responses in control and experimental fish. A significance level of *p* < 0.05 was used throughout.

## Results

For ANOVA analysis, the factors analyzed were the effects of tank pH (i.e., the pH at which the fish were kept: pH 7.7 or 8.2), odorant pH (i.e., the pH of the odorant solutions delivered to the olfactory epithelium: pH 7.7 or 8.2) and odorant concentration (10^−3^ to 10^−6^ M for the amino acids). Results are summarized in Table 3. While tank and odorant pH only showed significant effects for some of the odorants, the concentration always had a significant effect. Factors that showed an effect were further analyzed by linear regression or fit to a Hill three-parameter concentration-response curve.

**Table 3:** ANOVA analysis (Univariate analysis of variance, SPSS) for factors: tank pH, odorant pH, and odorant concentration on the detection of different odorants.

Odorant	Tank pH	Odorant pH	Odorant concentration
L-serine	*F*_95_ = 0.943, *p* = 0.335	***F*_95_ = 24.316, *p* < 0.0001**	***F*_95_ = 105.056, *p* < 0.0001**
L-leucine	*F*_103_ = 2,347, *p* = 0.996	***F*_103_ = 39.496, *p* < 0.0001**	***F*_103_ = 38.899, *p* < 0.0001**
L-arginine	*F*_103_ = 0.495, *p* = 0.110	***F*_103_ = 16.278, *p* < 0.0001**	***F*_103_ = 70.548, *p* < 0.0001**
L-glutamine	*F*_95_ = 3.225, *p* = 0.067	*F*_95_ = 0.835, *p* = 0.438	***F*_95_ = 53.78, *p* < 0.0001**
L-glutamate	***F*_95_ = 4.775, *p* = 0.002**	***F*_95_ = 29.08, *p* < 0.0001**	***F*_95_ = 158.742, *p* < 0.0001**
Bile fluid	*F*_95_ = 4.973, *p* = 0.09	*F*_95_ = 0.786, *p* = 0.46	***F*_95_ = 83.962, *p* < 0.0001**
Intestinal fluid	***F*_95_ = 4.276, *p* = 0.004**	*F*_95_ = 5.518, *p* = 0.06	***F*_95_ = 59.682, *p* < 0.0001**

Factors that have significant effects are mentioned in bold.

### Effects of Odorant pH on Olfactory Sensitivity in Control Fish

**Figure 1:**
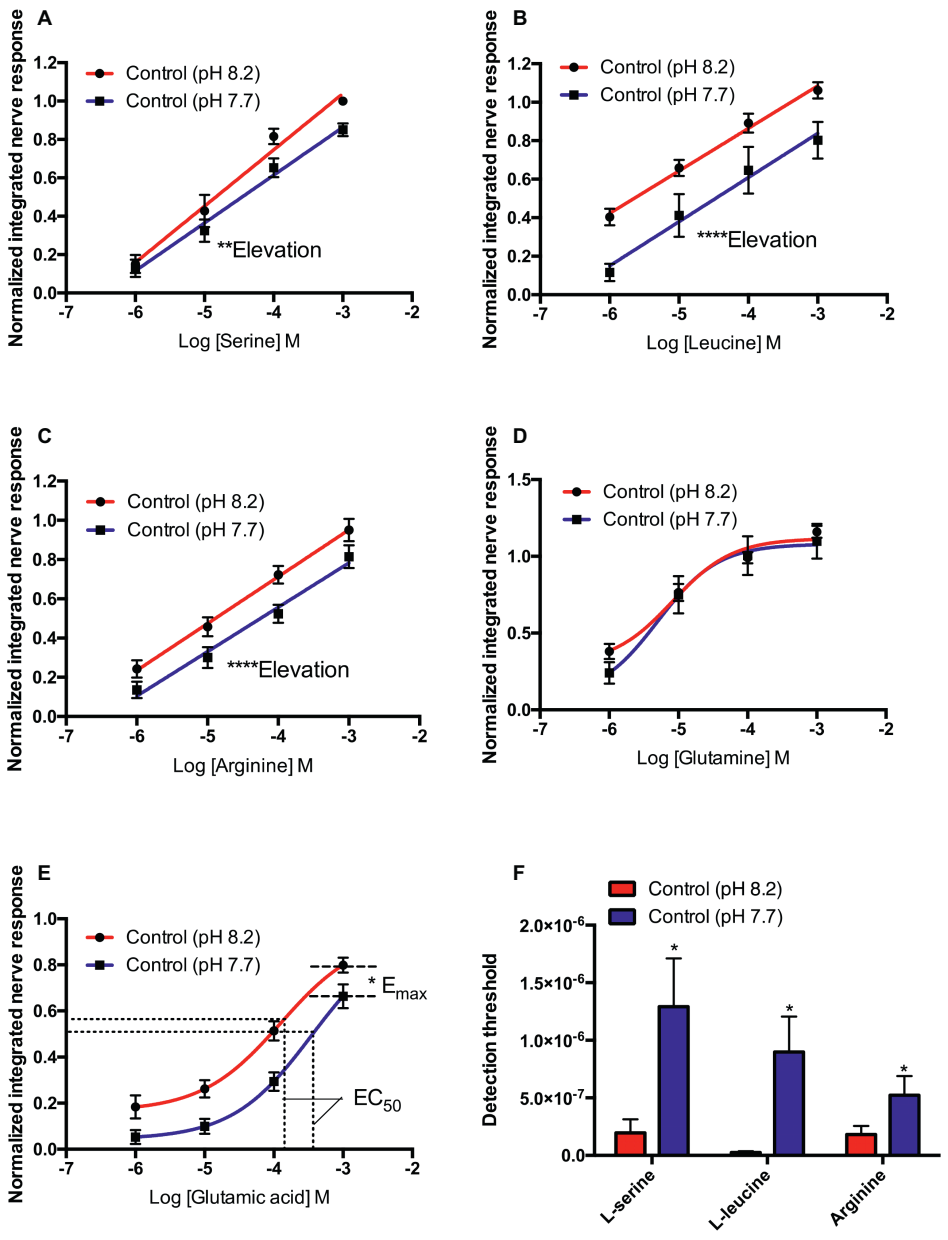
Normalized olfactory nerve responses of control fish (kept at pH 8.2) to **(A)** L-serine, **(B)** L-leucine, **(C)** L-arginine, **(D)** L-glutamine, and **(E)** L-glutamic acid; under control (red: odorant pH 8.2) and elevated *P*CO_2_ conditions (blue: odorant pH 7.7). **(F)** Effects of acute exposure to elevated *P*CO_2_ in the olfactory detection threshold of L-serine, L-leucine, and L-arginine. Values are shown as mean ± S.E.M. ^*^p < 0.05, ^**^p < 0.01, ^***^p < 0.001; *n* = 6.

In control seabream (kept at pH 8.2 prior to electrophysiological recording), the elevation of the concentration-response curves to L-serine (*F*_45_ = 9.363, *p* < 0.01), L-leucine (*F*_53_ = 25.225, *p* < 0.0001), and L-arginine (*F*_52_ = 0.1736, *p* = 0.68) tested at pH 8.2 was significantly higher than at pH 7.7. However, there were no differences between the slope of the concentration-response curves at pH 8.2 and 7.7 (Figures 1A–C). In addition, exposure to low pH water significantly increased the threshold of detection (Figure 1F) for L-serine (*T*_5_ = 3.170, *p* < 0.05), L-leucine (*T*_6_ = 2.848, *p* < 0.05), and L-arginine (*T*_6_ = 2.862, *p* < 0.05).

In contrast to L-serine, L-arginine, and L-leucine, the concentration-response curves of L-glutamine and L-glutamate did not fit linear regression well; the best-fitting model was the three-parameter Hill curve (Figures 1D,E). Olfactory nerve responses of control fish to L-glutamine at pH 8.2 and 7.7 were statistically equivalent (Figure 1D). There was no difference (*T*_5_ = 2.205, *p* = 0.08) between the EC_50_ for L-glutamate of control fish tested at pH 8.2 (1.142 × 10^−4^ M) and pH 7.7 (3.89 × 10^−4^ M), but the maximum response (*E*_max_) was significantly (*T*_5_ = 2.886, *p* < 0.05) higher at odorant pH 8.2 (0.88 ± 0.05) than at pH 7.7 (0.78 ± 0.05; Figure 1E).

**Figure 2:**
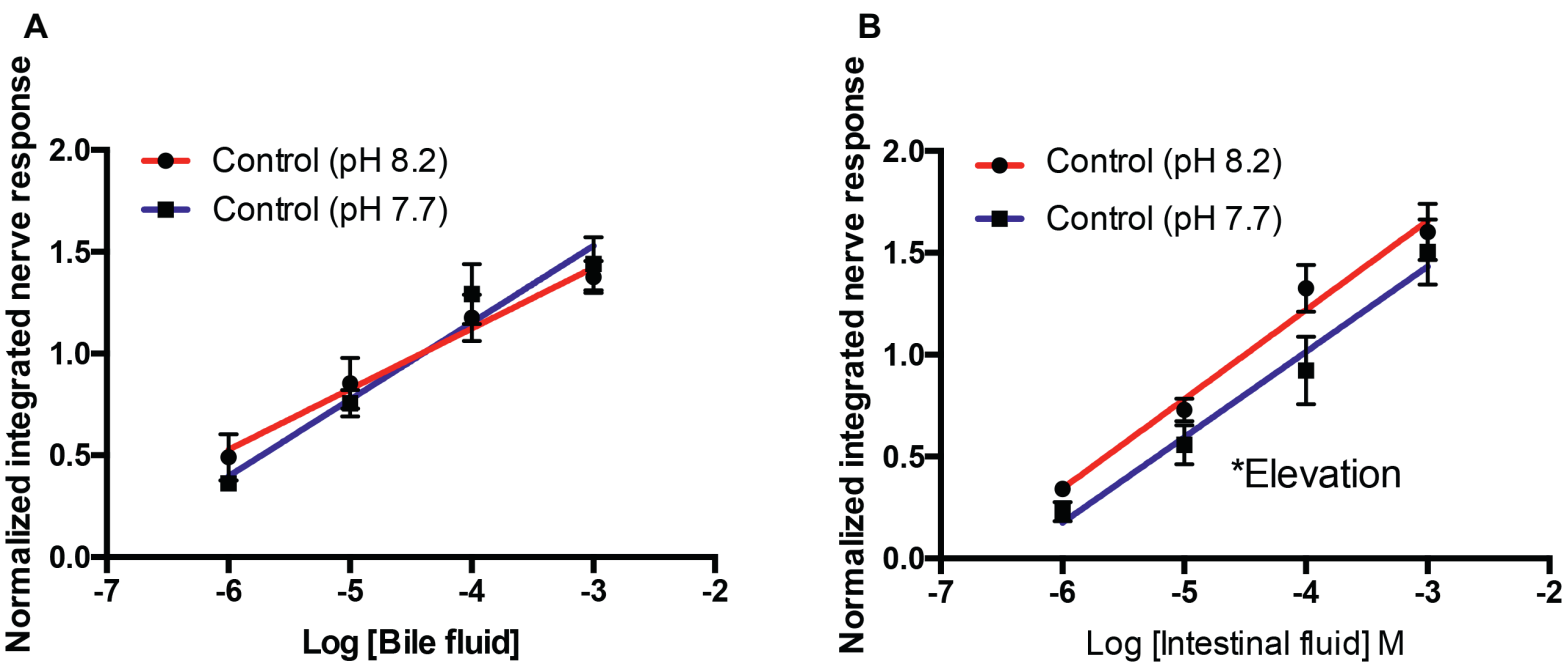
Normalized olfactory nerve responses of control fish (kept at pH 8.2) to conspecific bile fluid **(A)** and intestinal fluid **(B)** with odorant pH 8.2 (red) and pH 7.7 (blue). Values are represented as mean ± S.E.M; ^*^p < 0.05, *n* = 6.

For bile fluid, there were no differences in slope (*F*_36_ = 1.419, *p* = 0.24) or elevation (*F*_37_ = 0.022, *p* = 0.83) at pH 8.2 and 7.7 (Figure 2A). For intestinal fluid, the slope of the curves at pH 8.2 and 7.7 was not different (*F*_44_ = 0.072, *p* = 0.79); however, the elevation of the curves was statistically higher (*F*_45_ = 6.374, *p* < 0.05) at pH 8.2 than 7.7 (Figure 2B).

### Effect of Odorant pH on Olfactory Sensitivity in High CO_2_ Fish

**Figure 3:**
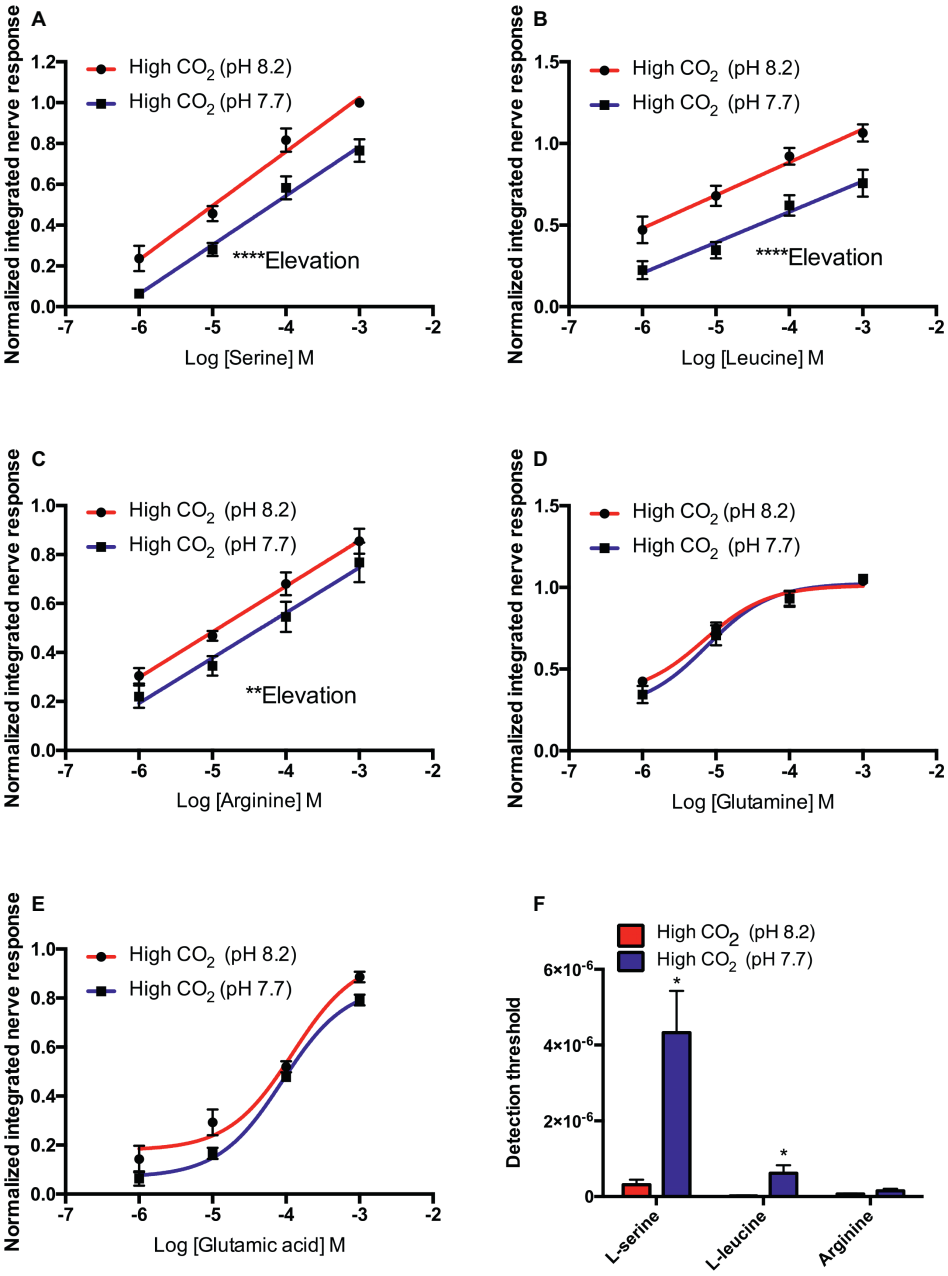
Normalized olfactory nerve responses of high CO_2_ fish (kept at pH 7.7) to **(A)** L-serine, **(B)** L-leucine, **(C)** L-arginine, **(D)** L-glutamine, and **(E)** L-glutamic acid; under odorant pH 8.2 (red) and odorant pH 7.7 (blue) conditions. **(F)** Effects of exposure to elevated *P*CO_2_ in the olfactory detection threshold of L-serine, L-leucine, and L-arginine. Values are shown as mean ± S.E.M. ^*^p < 0.05, ^**^p < 0.01, ^***^p < 0.001; *n* = 6.

As for controls, in high CO_2_ fish (kept at pH 7.7 for at least 4 weeks prior to electrophysiological recording) the elevation of the concentration-response curves to L-serine (*F*_45_ *=* 42.156, *p* < 0.0001), L-leucine (*F*_45_ = 46.971, *p* < 0.0001), and L-arginine (*F*_43_ = 9.888, *p* < 0.01) tested at pH 8.2 was significantly higher than at pH 7.7 (Figures 3A–C). However, there were no differences between the slopes of the concentration-response curves at pH 8.2 and 7.7 (Figures 3A–C). In addition, exposure to low pH water significantly increased the threshold of detection (Figure 3F) to L-serine (*T*_5_ = 3.464, *p* < 0.05) and L-leucine (*T*_5_ = 3.084, *p* < 0.05) but not to L-arginine (*T*_10_ = 1.578, *p* = 0.07).

As in the case of control fish, in the high CO_2_ animals the best-fitting model for the concentration-response curve to L-glutamine and L-glutamate was the three-parameter Hill curve (Figures 3D,E). Sensitivity of high CO_2_ fish to L-glutamine and L-glutamate at pH 8.2 and 7.7 were statistically equivalent; there were no differences between the EC_50_ or the *E*_max_ for these odorants (Figures 3D,E).

**Figure 4:**
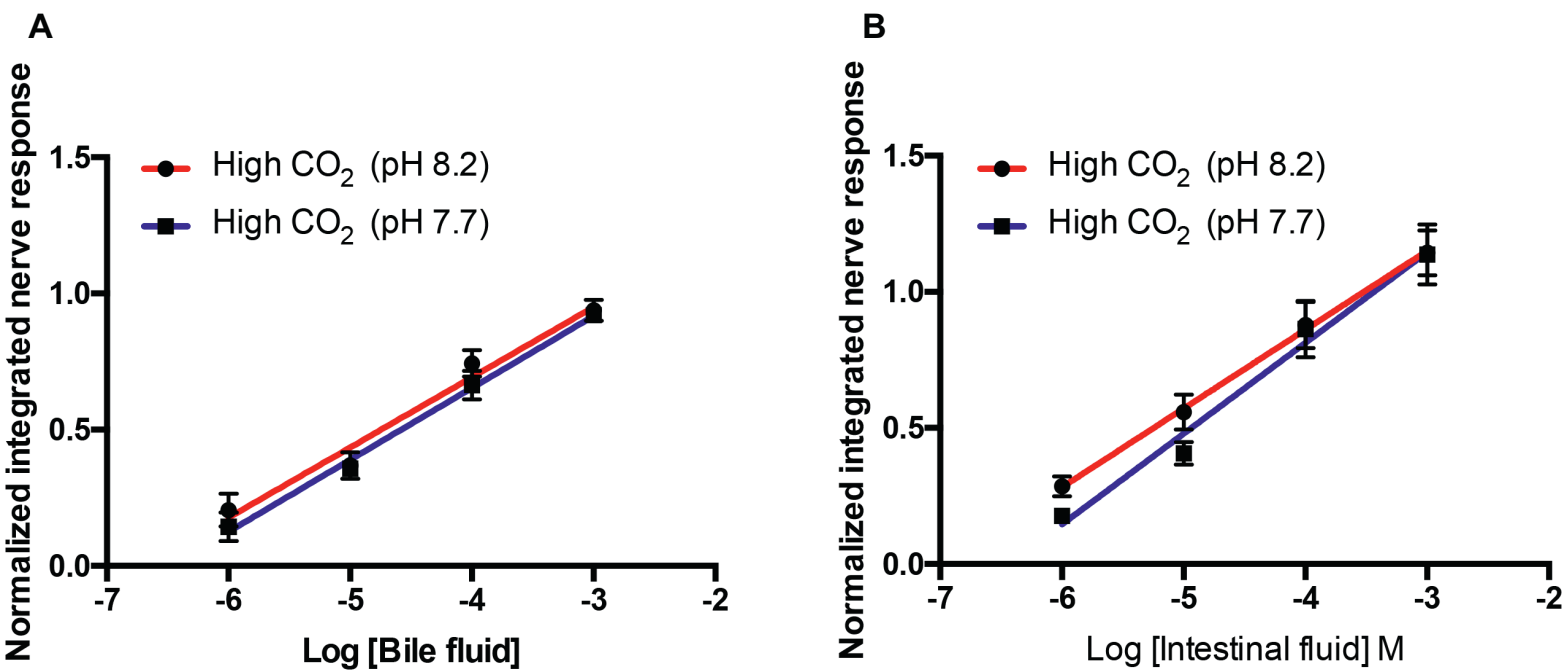
Normalized olfactory nerve responses of high CO_2_ fish (kept at pH 7.7) to conspecific **(A)** bile fluid and **(B)** intestinal fluid with odorant pH 8.2 (red) and pH 7.7 (blue). Values are shown as mean ± S.E.M; *n* = 6.

The olfactory nerve responses of high CO_2_ fish to bile fluid and intestinal fluid at pH 8.2 and 7.7 were statistically equivalent; there were no differences in the slope or elevation of the concentration-response curves (Figure 4).

### Short-Term (Acute) vs. Medium-Term Effects

**Figure 5:**
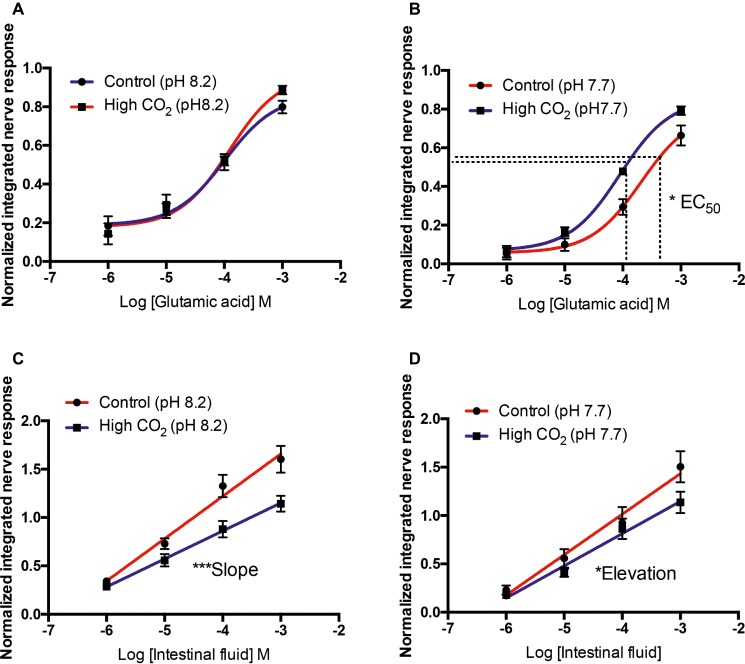
Normalized olfactory nerve responses of control and high CO_2_ fish to L-glutamate **(A,B)** and intestinal fluid **(C,D)** with the nostril conditioned with water pH 8.2 **(A,C)** and pH 7.7 **(B,D)**. Values are shown as mean ± S.E.M. ^*^p < 0.05; ^***^p < 0.001; *n* = 6.

When tested at odorant pH 8.2 or 7.7, responses to L-serine, L-leucine, L-arginine, L-glutamine, and bile fluid were similar in control and high CO_2_ fish; that is, medium-term exposure to high CO_2_ did not influence the effects of acute changes in odorant pH. As there were significant effects of medium-term exposure to high *P*CO_2_ on the olfactory response to L-glutamic acid and intestinal fluid (Table 3), we compared responses of control and high CO_2_ fish at the same odorant pH. Comparing sensitivity to L-glutamic acid between control and high CO_2_ fish at odorant pH 8.2 showed that there were no significant differences between EC_50_ (*T*_9_ = 0.543, *p* = 0.60) or *E*_max_ (*T*_9_ = 1.814, *p* = 0.10) (Figure 5A). At odorant pH 7.7, however, the EC_50_ of control fish was significantly (*T*_10_ = 2.357, *p* < 0.05) higher than that of high CO_2_ fish. There was no difference between the *E*_max_ (*T*_10_ = 1.317, *p* = 0.22) of control and high CO_2_ fish (Figure 5B).

For intestinal fluid at odorant pH 8.2, differences between the slopes of control and high CO_2_ fish were highly significant (*F*_44_ = 8.15, *p* < 0.01); it was therefore not possible to compare the intercepts (Figure 5C). For odorant pH 7.7, there was no differences in the slope (*F*_44_ = 1.70, *p* = 0.20) of control and high CO_2_ fish; however, the elevation was significantly different (*F*_45_ = 4.51, *p* < 0.05); the controls were more sensitive than the high CO_2_ fish (Figure 5D).

### Comparison of pH-Induced Effects on Protonation State and Olfactory Sensitivity

The effect of high CO_2_ water on the olfactory sensitivity was reversed by exposing the olfactory epithelium to normal seawater for 10 min, with the exception of the decrease in amplitude of response to intestinal fluid seen in high CO_2_ fish. The transient nature of the observed changes in olfactory sensitivity at low pH, and that the extent of the changes depended on the odorant, raised the hypothesis that the mechanism involved is related to pH-driven changes in protonation of odorants and/or receptor(s), due to the reduction in water pH. To test this hypothesis, and understand the real impact of pH on olfactory sensitivity, the proportion of each protonation state over the pH range was calculated based on the pK_a_ using the Henderson-Hasselbalch equation. Assuming that the protonation state of the ligand (odorant) and/or the binding site of its receptor affect(s) binding affinity (reviewed in Strasser et al., 2017), we calculated the stimulus concentrations at different states of protonation (Table 4).

**Table 4:** Calculated effective stimulus concentrations during bioassays at pH 8.2 and 7.7 as well as the respective change in stimulus concentration in percentage between pH-conditions.

Amino acid	Stimulus concentration used during bioassay (M)	Effective stimulus concentration at pH 8.2 (M)	Effective stimulus concentration at pH 7.7 (M)	Change in effective stimulus concentration
L-Serine	10^−6^ to 10^−3^ M	8.90 × 10^−8^ to 10^−5^	3.00 × 10^−8^ to 10^−5^	−33.7%
L-Leucine	10^−6^ to 10^−3^ M	4.00 × 10^−8^ to 10^−5^	1.30 × 10^−8^ to 10^−5^	−32.5%
L-Arginine	10^−6^ to 10^−3^ M	13.7 × 10^−8^ to 10^−5^	4.77 × 10^−8^ to 10^−5^	−34.8%
L-Glutamine	10^−6^ to 10^−3^ M	10.5 × 10^−8^ to 10^−5^	3.58 × 10^−8^ to 10^−5^	−34.1%
L-Glutamate	10^−6^ to 10^−3^ M	5.10 × 10^−8^ to 10^−5^	1.67 × 10^−8^ to 10^−5^	−32.7%

Concentrations were tested in steps of 10^−1^ within the range indicated.

These values were introduced into the linear regression or three-parameter Hill equation obtained experimentally from the response to odorants at a pH 8.2. The resulting points were plotted to simulate the extent of change caused by stimulus protonation state and compared with the extent of change observed for the olfactory nerve response at pH 7.7.

**Figure 6:**
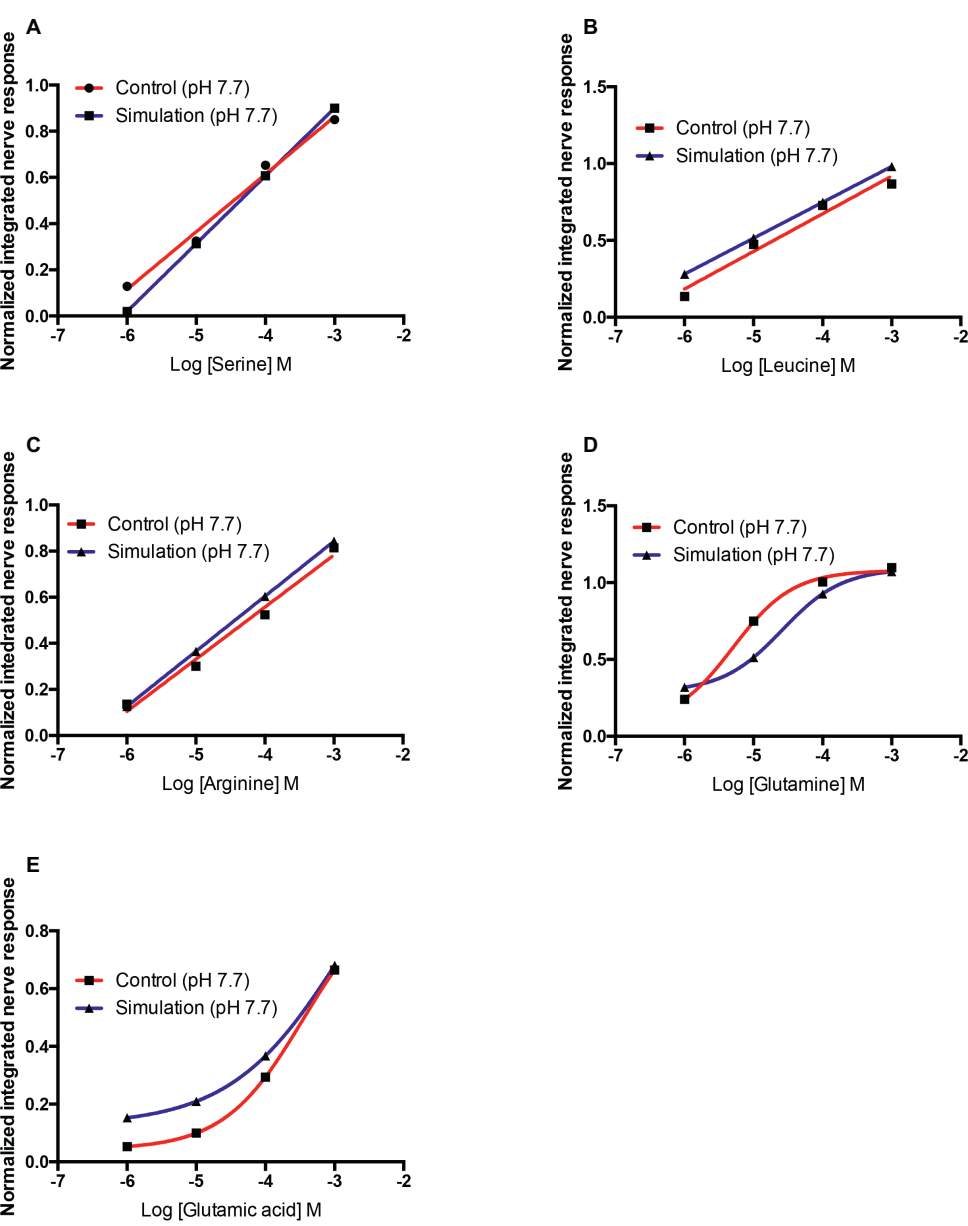
Normalized olfactory nerve responses of control fish (red) with the nostril conditioned with water pH 7.7 and expected response (blue), based on the effective odorant concentration at pH 7.7 and the linear regression equation or three-parameter Hill equation fit **(A)** L-serine, **(B)** L-leucine, **(C)** L-arginine, **(D)** L-glutamine, and **(E)** L-glutamic acid. Values are shown as mean ± S.E.M.

For all amino acids tested, a reduction in pH caused a reduction in the concentration of non-protonated ligand. The concentrations of active ligand at pH 7.7 were 32–35% lower than at pH 8.2 (Table 4). For L-serine, L-leucine, and L-arginine, the reduction in the olfactory response recorded was similar to that predicted by the theoretical protonation model (Figures 6A–C). However, for L-glutamine, a decrease in olfactory sensitivity was predicted (Figure 6D) but there was little observed difference between pH 8.2 and 7.7. Finally, for L-glutamic acid the decrease in the olfactory nerve response was lower than predicted by the protonation model (Figure 6E).

**Figure 7:**
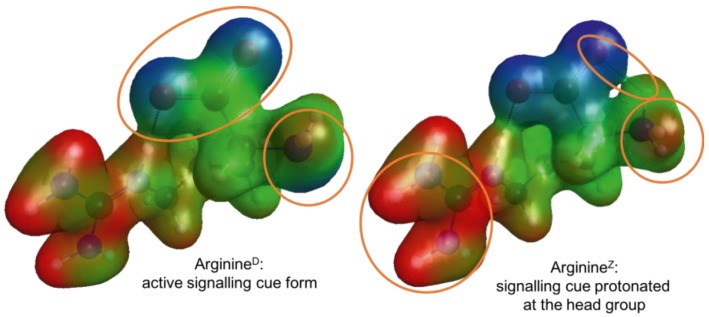
Active (Arginine^D^, left) and protonated (Arginine^Z^, right) conformers of L-arginine with their molecular electrostatic potential mapped onto an iso-electron density surface. Negative charge is colored in blue, neutral in green, and positive charge in red. The orange circles highlight differences in the charge distribution between the two conformer models.

It is well-known that pH affects the protonation state of amino acids. This protonation changes the overall molecular charge and the charge distribution, which could affect interaction of the odorant with its receptor. To assess this, quantum chemical calculations were performed to obtain the energetically most favorable conformers and then the charge distribution for the two relevant protonation states of L-arginine were obtained (Figure 7) as an example. While the positive charge (red) of the side chain remains, the distribution of the negative charge (blue) around the head group shifts with protonation of the amino group.

### Histology

**Figure 8:**
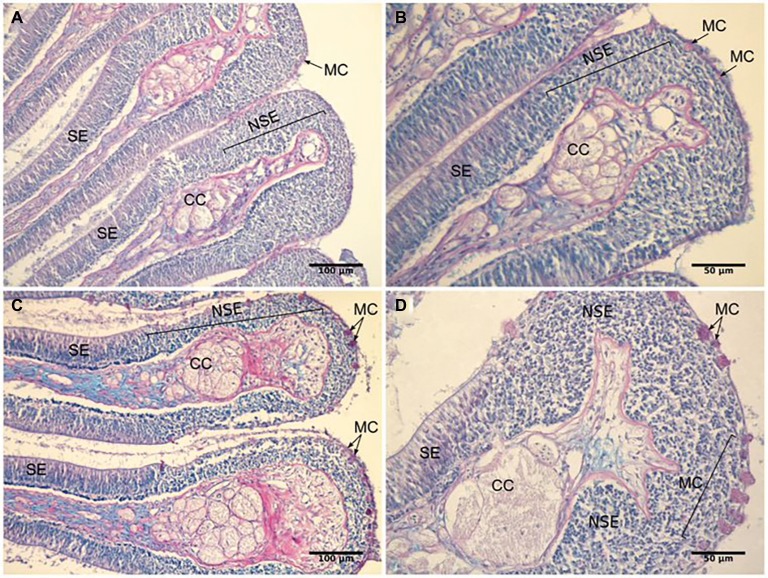
Representative histological sections of the olfactory lamellae of the gilthead seabream stained with periodic acid-Schiff/Alcian blue to show the effects of exposure to high *P*CO_2_/low pH on tissue morphology. **(A,B)** control fish; **(C,D)** high CO_2_ fish. CC, Central core; MC, Mucus cell; NSE, non-sensory epithelium; SE, sensory epithelium.

The olfactory epithelia of fish kept for 4–8 weeks in water of pH 7.7 showed pronounced morphological changes, including an increase in the area of the non-sensory epithelium, and an increase in the number of mucus cells. The oval olfactory rosette (OR) consists of 32–38 lamellae (OL) that radiate from a median raphe (MR). There were no marked differences in the gross anatomy of the olfactory organ between fish kept in control and high CO_2_ water. Each lamella has two layers of epithelium that enclose the central core (CC). The central core is separated from the epithelium by the basement membrane (BM). The non-sensory area consists of a stratified epithelium covering the outer margins of the lamella (NSE). The sensory area consists of pseudostratified epithelium covering lateral sides of the lamella (SE) (Figures 8C,D).

In high CO_2_ fish (pH 7.7), the ratio between non-sensory and sensory epithelium (0.34 ± 0.02) is significantly higher (*T*_8_ = 5.583*, p* < 0.001) than in controls (0.21 ± 0.01). Furthermore, the number of mucus cells *per* lamellae was significantly higher (*T*_8_ = 3.786, *p* < 0.01) in high CO_2_ fish (21.6 ± 1.7) than controls (11.8 ± 2.0) (Figure 8). Interestingly, mucus cells are present only in the nonsensory epithelium; the increased number of these cells in high CO_2_ seabream may be related to the augmented area of non-sensory epithelium. In addition, to the higher number of mucus cells, the color of these cells, when examined for neutral and acidic mucus by combined Periodic Acid-Schiff (PAS)-Alcian blue staining, was deep pink in high CO_2_ fish and colorless or light pink in controls (Figure 8); these differences suggest a shift in pH of the mucus from acidic to more neutral.

## Discussion

The current study shows that, in the seabream, increased oceanic *P*CO_2_ and/or reduction of pH predicted for the end of the century cause(s) a significant reduction of olfactory sensitivity to some, but not all, odorants. Significantly, this reduction persisted in seabream kept in high *P*CO_2_ water for several weeks; fish were unable to compensate. The effect of exposure to high CO_2_/low pH was complex and varied with the odorant type. The change in olfactory sensitivity can be partly – but not entirely – explained by a shift in the protonation state of the odorant molecule. Moreover, the current study shows that after several weeks exposure to high *P*CO_2_ there were morphological changes in the olfactory epithelium indicative that exposure to high CO_2_/low pH evokes a structural re-organization.

Overall, acute exposure of the olfactory epithelium to high CO_2_/low pH water reduced the olfactory response for five out of seven odorants tested, and increased the detection threshold in three out of those five, similar to previously reported in bass (Porteus et al., 2018). Importantly, however, the current study also showed that this effect persisted, even after 4 weeks exposure to acidified water. There was an increase in the threshold of detection and a decrease in the amplitude of responses to L-serine, L-leucine and L-arginine, but not to bile fluid. This means that the fish would have to be closer to an odor source in order to detect it. This would have obvious repercussions in food-search and predator-avoidance behaviors, but may also help explain disrupted olfactory-guided homing and/or migration (Munday et al., 2009; Devine et al., 2012a). There were differences in the amplitude of response to intestinal fluid, but the detection threshold did not change. As intestinal fluid is likely a mixture of different odorants, this may reflect a reduction in all or just some; if the latter, then this may imply a qualitative effect on olfactory sensitivity, as well as a quantitative effect. When tested at odorant pH 8.2 or 7.7, responses to L-serine, L-leucine, L-arginine, L-glutamine, and bile fluid were similar in control and high CO_2_ fish. This suggests that medium term exposure to high CO_2_ does not cause changes in the olfactory sensitivity to these odorants *per se*; rather, the seabream is unable to compensate for the effect of low pH/high *P*CO_2_ on acute sensitivity. However, at odorant pH 7.7, olfactory sensitivity to glutamate was higher in high CO_2_ fish than in controls, suggesting that, in this specific case, the sensitivity to L-glutamate can somehow acclimate to high CO_2_/low pH. This may involve changes in the expression and/or type of receptor(s) in the olfactory epithelium; downregulation, but not upregulation of olfactory receptors was seen in the bass (Porteus et al., 2018), whereas no such changes were seen in the salmon (Williams et al., 2019). In contrast, the sensitivity to intestinal fluid when tested in seawater at pH 8.2, was higher in control fish than in high CO_2_ fish. This suggests that medium-term exposure to CO_2_ impairs the olfactory detection of intestinal fluid and, unlike the other stimuli tested, this was not reversible by bathing the olfactory epithelium with normal seawater. Again, this is likely to reflect a change in receptor expression. Thus, the effect of increased *P*CO_2_ on olfactory sensitivity varies with the odorant type.

The sensitivity to some odorants decreased as soon as the olfactory epithelium was exposed to high CO_2_ water; this effect occurred in both control and high CO_2_ fish and was reversed by bathing the epithelium with seawater at pH 8.2. We propose that the effect can be directly linked to the effects of pH on the odorant molecules and/or their receptors in fish as well as crabs (Roggatz et al., 2016). We show the changes in charge distribution can alter the conformation of protonated L-arginine, for example. Changes in protonation state are reversible and much more rapid (<micro-seconds) than any physiological and/or behavioral change which may take several days (for example, see Munday et al., 2009, 2010; Hamilton et al., 2014). Olfactory sensitivity in vertebrates, including fish, is mediated by G-protein coupled receptors (GPCRs) (Buck and Axel, 1991; Ngai et al., 1993). Changes in pH have been shown to alter GPCR-ligand affinity *in vitro* (Ruan et al., 2009), and charged amino acid residues in the receptor play multiple roles in receptor-ligand interaction (Strasser et al., 2017).

Another effect of medium term exposure to high CO_2_ water was the reduction of olfactory sensitivity to odorants in intestinal fluid. This may be due to qualitative and/or quantitative changes in the olfactory epithelium, as observed in the present study, which we hypothesize may cause a change in the expression of some odorant receptors (as in the bass; Porteus et al., 2018). Finally, in the case of L-glutamate, the decrease in olfactory sensitivity in high CO_2_ water was lower in animals chronically exposed than in those acutely exposed. This may show a degree of adaptation to high *P*CO_2_ and may be a result of morphological re-organization and/or changes in olfactory receptor expression. Further studies are required to evaluate whether exposure to high CO_2_ water induces changes in gene expression in the olfactory epithelium. Nonetheless, it should be noted that it is difficult to separate the direct effect of increased *P*CO_2_ and the consequent reduction in pH (Ou et al., 2015). A recent study in coho salmon (Williams et al., 2019), while showing that increased *P*CO_2_ affects both olfactory-mediated behavior and gene expression in the olfactory system, suggested that olfactory sensitivity is unaffected by increased *P*CO_2_/low pH at the level of the olfactory epithelium. However, this study used a different experimental approach (i.e., olfactory responses at different *P*CO_2_/pH levels were not compared in the same fish), and the high concentration (10^−2^ M) of odorants used, coupled with the lower sensitivity of the electro-olfactogram (EOG) when used in seawater (Silver et al., 1976; Hubbard et al., 2011) makes this negative finding difficult to interpret.

### Does Exposure to High *P*CO_2_ Modify the Olfactory Epithelium Organization?

Medium term (4 weeks) exposure to high CO_2_ induced an increase in the ratio between nonsensory vs. sensory epithelium and an augmentation in the number of mucus cell per lamellae. Furthermore, there was a shift in pH of the mucus from acidic to more neutral. This suggests that high CO_2_ seawater evokes modification of the epithelium and causes increased proliferation of neutral mucus-producing goblet cells to reinforce the outer extrinsic barrier. This may be an adaptive response, as the mucus will protect the underlying epithelial cells, including the olfactory receptor neurones, but the charge, viscosity and the thickness of the layer may also reduce the odorant/receptor interaction and thus lower sensitivity (Rygg et al., 2013; Corfield, 2015). However, the olfactory sensitivity of seabream exposed for 4 weeks to high *P*CO_2_ was similar to that of controls, when tested at the same pH. The enlarged area of non-sensory epithelium might also represent an adaptation to high CO_2_; an augmented area of non-sensory epithelium positioned in the upper and outer margin of the olfactory lamellae results in a localization of the sensory area deeper in the olfactory lamellae, and as a consequence it is less exposed. On the other hand, it could represent impairment of olfactory epithelium formation, growth and/or renewal, and ultimately it might contribute to decreased olfactory sensitivity given that the sensory area is reduced. Initial studies clearly indicate changes in gene expression induced by ocean acidification in the olfactory system (Porteus et al., 2018; Williams et al., 2019). Further studies are required to evaluate the mucin forms expressed, the factors driving the increased number of goblet cells, necrosis and/or apoptosis of the epithelium and changes in the number, type and distribution of olfactory receptor neurones and/or receptors and ion pumps and channels within the olfactory epithelium.

### Does Seawater pH Change the Protonation of Odorant Molecules and Thereby Modify Olfactory Sensitivity?

The transient nature of the observed changes in olfactory sensitivity raised the hypothesis it is due to changes in the protonation state of odorant and/or receptor, due to changes in water pH, as previously suggested for the crab pheromone (Roggatz et al., 2016). Fish odorants are mostly low molecular weight organic molecules with high water solubility (Laberge and Hara, 2004), such as amines (Rolen et al., 2003), amino acids, nucleotides, nucleosides and organic acids (reviewed in Hara, 1994). Amino acids contain both functional groups attached to the same carbon (α-carbon) and the various alpha amino acids differ in the side chain attached to the α-carbon. Amino acid detection by the olfactory epithelium probably involves different receptors that are relatively ligand-specific and for activation require specific molecular features (Friedrich and Korsching, 1997, 1998; Fuss and Korsching, 2001). Our hypothesis is that change of pH in future oceans could lead to changes in protonation states of amino acids (and other odorants) which, in turn, may lead to significant alterations in their structure and affinity for their receptor(s). Herein, we determined that the difference in pH from 8.1 to 7.7 would reduce the concentration of non-protonated amino acids by up to 35%. This coincided with a reduction in olfactory response, except for L-glutamine. This may be because, for L-glutamine, the binding site of the receptor is not dependent on the protonation state of the ligand. Furthermore, for L-glutamic acid, reductions in olfactory responses were greater than predicted by the change in protonated/non protonated ratio. This could be explained by the occurrence of changes of charge distribution within the binding site of the receptor (Tierney and Atema, 1988; Strasser et al., 2017); thus, conformational changes in both odorant and receptor may contribute to the decrease in olfactory sensitivity. However, other mechanisms – such as effects on the transduction pathways (Rong et al., 2018) – may also play a role.

In conclusion, the current study shows that predicted future oceanic pH will influence fish olfactory sensitivity by acting directly on the olfactory epithelium. Exposure to high CO_2_ water decreased olfactory nerve responses to most of the odorants tested. Furthermore, seabream were unable to compensate for high CO_2_ levels and, even after 4 weeks exposure to high, the olfactory sensitivity remained reduced. Our hypothesis is that changes of charge, structure and consequently function of odorant molecules and/or their receptors due to pH change reduces receptor-ligand affinity, as has previously been shown in crabs (Roggatz et al., 2016). Thus, we propose that this is a purely physicochemical issue and explains why fish are unable to compensate. However, changes in the protonation state of odorants is not the only mechanism involved in olfactory disruption due to high CO_2_ water; long exposure of seabream led to morphological changes in the olfactory epithelium and diminution of olfactory sensitivity to intestinal fluid. Importantly, the protonation effect and the modification of epithelial structure presumably act in combination with the reported alterations in brain neurotransmitter function (Nilsson et al., 2012) and suggest the increase in CO_2_ in seawater acts at multiple levels in sensory systems and will give rise to complex and diverse outcomes (Kelley et al., 2018). Further studies are required to evaluate whether changes in olfactory receptor expression or modifications in the olfactory epithelium are contributing to the long term effects of CO_2_ on the olfactory sensitivity of seabream and, probably, other fish.

## Data Availability

The datasets generated for this study are available on request to the corresponding author.

## Ethics Statement

Animal maintenance and experimentation was carried out in certified experimental facilities and followed Portuguese national legislation (DL 113/2013) under a “group-1” license by the Veterinary General Directorate, Ministry of Agriculture, Rural Development and Fisheries of Portugal.

## Author Contributions

ZV, JH, and PH contributed to conception and design of the study. ZV and PH designed and carried out the electrophysiology experiments. CR and DB designed and carried out the chemical modeling. ZV did the histology. ZV, CR, and PH wrote the first draft of the manuscript. All authors contributed to manuscript revision, read, and approved the submitted version.

### Conflict of Interest Statement

The authors declare that the research was conducted in the absence of any commercial or financial relationships that could be construed as a potential conflict of interest.
